# Biomarkers of browning of white adipose tissue and their regulation during exercise- and diet-induced weight loss^1^,^2^

**DOI:** 10.3945/ajcn.116.132563

**Published:** 2016-08-03

**Authors:** Asif Nakhuda, Andrea R Josse, Valentina Gburcik, Hannah Crossland, Frederic Raymond, Sylviane Metairon, Liam Good, Philip J Atherton, Stuart M Phillips, James A Timmons

**Affiliations:** 3 School of Medicine, Derby Royal Hospital, University of Nottingham, Nottingham, United Kingdom;; 4 Department of Kinesiology, Brock University, St. Catharines, Canada;; 5 Royal Veterinary College, London, United Kingdom;; 6 Division of Genetics and Molecular Medicine, King’s College London, London, United Kingdom;; 7 Functional Genomics, Nestle Institute of Health Sciences, Lausanne, Switzerland; and; 8 Exercise Metabolism Research Group, McMaster University, Hamilton, Canada

**Keywords:** browning, exercise, microarray, obesity, weight loss, white adipose tissue

## Abstract

**Background:** A hypothesis exists whereby an exercise- or dietary-induced negative energy balance reduces human subcutaneous white adipose tissue (scWAT) mass through the formation of brown-like adipocyte (brite) cells. However, the validity of biomarkers of brite formation has not been robustly evaluated in humans, and clinical data that link brite formation and weight loss are sparse.

**Objectives:** We used rosiglitazone and primary adipocytes to stringently evaluate a set of biomarkers for brite formation and determined whether the expression of biomarker genes in scWAT could explain the change in body composition in response to exercise training combined with calorie restriction in obese and overweight women (*n* = 79).

**Design:** Gene expression was derived from exon DNA microarrays and preadipocytes from obesity-resistant and -sensitive mice treated with rosiglitazone to generate candidate brite biomarkers from a microarray. These biomarkers were evaluated against data derived from scWAT RNA from obese and overweight women before and after supervised exercise 5 d/wk for 16 wk combined with modest calorie restriction (∼0.84 MJ/d).

**Results:** Forty percent of commonly used brite gene biomarkers exhibited an exon or strain-specific regulation. No biomarkers were positively related to weight loss in human scWAT. Greater weight loss was significantly associated with less uncoupling protein 1 expression (*P* = 0.006, *R*^2^ = 0.09). In a follow-up global analysis, there were 161 genes that covaried with weight loss that were linked to greater CCAAT/enhancer binding protein α activity (*z* = 2.0, *P* = 6.6 × 10^−7^), liver X receptor α/β agonism (*z* = 2.1, *P* = 2.8 × 10^−7^), and inhibition of leptin-like signaling (*z* = −2.6, *P* = 3.9 × 10^−5^).

**Conclusion:** We identify a subset of robust RNA biomarkers for brite formation and show that calorie-restriction–mediated weight loss in women dynamically remodels scWAT to take on a more-white rather than a more-brown adipocyte phenotype.

See corresponding editorial on page 545.

## INTRODUCTION

There are ≥3 phenotypic versions of the adipocyte in mammals (1, 2). The classic brown adipocyte, which originates from a myogenic lineage (1, 3), can express high amounts of uncoupling protein 1 (*UCP1*)^9^ and forms brown adipose tissue, which is a tissue that is physiologically adapted for heat production through the uncoupling of respiration. In contrast, white adipocytes are derived from a lineage that is distinct from brown adipose tissue (4) and do not typically express *UCP1*. Nevertheless, cells with biochemical features of brown adipocytes can be present in white adipose tissue (WAT) depots (5, 6), and these depots have been termed brite (brown-in-white) or beige adipocytes (7). Brown and brite adipocytes have the potential to increase energy expenditure (8) through the activation of UCP1, and increased *UCP1* messenger RNA (which is a biomarker for brite or brown cells) is associated with a healthier metabolic phenotype in obese humans (9). Thus, it has been proposed that brite formation (with drugs) could be an effective treatment of metabolic disease in humans. Although this concept is appealing, the long-term efficacy of increasing brite formation will depend on complex interactions between pharmacodynamics, appetite, and energy balance. Exercise training can promote a loss of body adipose tissue mass, and it has been proposed that this loss may be due to brite formation (10) and be part of the antidiabetic action of exercise (11). Similar to the situation of a negative energy balance encountered during cold stress in mice (12), it is plausible that the neuroendocrine recruitment of UCP1 activity occurs after an exercise-related negative energy balance. Nonetheless, to our knowledge, there have not been any previous larger-scale clinical studies that have confirmed this proposition, and the global molecular profile of human adipose tissue after exercise training or weight loss has also been understudied. To facilitate a successful pursuit of these concepts, novel molecular targets to promote the formation of brite cells and more-extensively validated biomarkers are required.

Rosiglitazone, which is a peroxisome proliferator–activated receptor-γ (PPARγ) agonist (13), is one of the few drugs used in humans that have been proven to generate brite adipocytes from white preadipocytes and even from the most developmentally white of adipose depots (epididymal) (2, 12), thereby suggesting that the PPARγ agonism is central to brite formation (14). Studies of how rosiglitazone mediates brite formation from white preadipocytes (2) have relied on a focused set of molecular markers (15) with limited technical validation. To extend this work, we used exon DNA microarray data (16) to better evaluate which RNA biomarkers of brite formation are likely to be most useful. Exon-based DNA microarray data have originated from mouse strains with a differential propensity for obesity (Sv/129 and C57BL/6). An analysis of the global transcriptome response to rosiglitazone could also potentially identify non-PPARγ ligands that might also promote brite formation (17). We applied a subset of stringent brite biomarkers to a large human study of subcutaneous white adipose tissue (scWAT) before and after exercise combined with modest calorie restriction to induce fat loss to determine the relation between weight loss and the regulation of genuinely stringent brite marker in vivo in humans.

## METHODS

### Human tissue and DNA microarray experiment data

Participants for our human study were from our previously published Improving Diet, Exercise and Lifestyle for women study (18, 19) (clinicaltrials.gov; NCT00710398) that was approved by the Research Ethics Board of the Hamilton Health Sciences and conformed to the most-recent Canadian government tricouncil funding policy statement on the use of human subjects in research. Biopsies were obtained from 85 overweight subjects before and after 16 wk of exercise training coupled with an ∼20% reduction in daily energy requirements of which 79 subjects had before and after body-composition measurements and gene-chip data (**Supplemental Table 1**). The primary outcome of this clinical study was to investigate the effect of different dairy and protein diets with exercise and showed that higher protein, with high dairy, was modestly beneficial to body composition in terms of a gain in lean mass and a loss of fat mass (18, 19). Regardless of the diet group, in the current molecular analysis, the groups were combined.

Briefly, each subject trained for 5 d/wk under supervision and for 2 additional days unsupervised. Needle biopsies (taken with the use of a 5-mm Bergstrom) were taken from scWAT just above the vastus lateralis muscle of the quadriceps before and after the training intervention. RNA was extracted with the use of an Agencourt RNAdvance Tissue Kit (Beckman Coulter). A total of 300 ng was amplified with the use of an Illumina TotalPrep-96 RNA amplification kit (Life Technologies), hybridized to a HumanHT-12 V4.0 Expression BeadChip (Illumina), and scanned with the use of a BeadArray Reader (Illumina) (at Nestle Research Center). Samples from 79 subjects passed standard quality-control methods. All microarray analysis was carried out using R (version 3.2.2). Raw array data were normalized with the use of quantile normalization, and detection calls were used to filter the chip data (without background subtraction). A median *P* value of 0.1 was used for detection rates across presamples and postsamples, which left 18,747 genes in the analysis (yielding a detected-gene count that was similar to the count in previous studies). Quantitative significance analysis of microarrays [using the SAMR package (Stanford University) in R, where the quantitative option in false discovery rate (FDR) adjusted correlative model] was implemented to generate a list of genes that vary in a positive or negative manner with weight loss (FDR <10%). Pearson correlation coefficients were calculated independently. Microarray data were deposited in the Gene Expression Omnibus database as GSE58559 (National Center for Biotechnology Information).

### Cell culture, drug treatment, and RNA profiling

Subcutaneous inguinal preadipocytes were isolated from Sv/129 or C57BL/6 mice (Harlan) and processed as previously described (20–22). Where indicated, cells were supplemented with or without 1 μmol rosiglitazone maleate/L (Enzo Life Sciences) from the first day of culture (20). RNA was extracted on day 7 with the use of TRizol solution (Life Technologies) as previously described (1). RNA was dissolved in 20 μL RNAse-free water and stored at −80°C for subsequent analysis as previously described (16). Sets of independent adipocytes cultures were produced for RNA for use in the reverse transcriptase–quantitative polymerase chain reaction (RT-qPCR) studies. A total of 500 ng total RNA was reversed transcribed with a High-Capacity cDNA Reverse Transcription kit (Life Technologies). Complementary DNA was diluted 1:5, and 1μL was added per well of the 384-optical well plates (Life Technologies). Primers were mixed with SYBR Select Master Mix (Life Technologies) and were run in triplicate. Thermal-cycling conditions were 2 min at 50°C followed by 2 min at 95°C and 40 cycles of 15 s at 95°C with 60 s at 60°C on a ViiA 7 Real-Time PCR System (Life Technologies). A eukaryotic 18S ribosomal RNA kit (Life Technologies) was used as an input control, and quantification relied on the Δcycle threshold method with primers for *Ucp1* and fatty acid binding protein 4 (*Fabp4*) as previously described (20, 21).

### Exon DNA microarray production

Twenty Affymetrix Murine Exon 1.0 ST arrays (Affymetrix) were processed as previously described (16). Three samples were excluded as outliers by normalized unscaled SE plots (supported by a principal component analysis) before any downstream statistical analysis was conducted. The raw data have been deposited in the Gene Expression Omnibus database as GSE57903, and these data have been used to develop a novel bioinformatic method for analyzing gene splicing (16). An update of the annotation of the exon array resulted in 16% of probes being removed because of cross-hybridization at multiple genomic loci. Subsequently, 10% of probe sets were removed because the signal was approximately background across all samples. Finally, the remaining probe sets (74% of all core probe sets) were mapped to a unique gene identifier and significance analysis of microarrays was used to estimate rosiglitazone-mediated differentially expressed genes in Sv/129 and C57BL/6 cells. To allow for an accurate meta-analysis of brite phenotypic adipocyte markers between our study (Sv/129 or C57BL/6 mice) and published data from studies using Naval Medical Research Institute (NMRI) mice (15), microarray probe sets were selected to precisely match the regions spanned by primers in the mentioned study (15) by using the Primer-BLAST tool (http://www.ncbi.nlm.nih.gov/tools/primer-blast/).

### Biological pathway analysis and upstream regulator analysis

Regulated genes used for pathway and gene ontology analysis need to be adjusted for technology and biological bias (23). To partially address this, the group of regulated genes was contrasted with all detectable genes in the experiment (and not with all genes on the gene chip or in the network database). An ingenuity pathway analysis (IPA) was used to identify potential upstream regulators of the regulated gene list, whereby regulated and background detectable genes were mapped to an IPA database (17). We used an upstream analysis whereby a *P* value was generated on the basis of the degree of overlap between a given gene set within the IPA database (which typically reflects the published gene changes in response to a range of transcription factors or drugs) and the differentially expressed genes in the current data. After adjusting for the data-set size, a Fisher’s exact test was applied. A second variable [activation or inhibition (*z* score)] was used to compare the directional change in the experimental gene set compared with in the IPA database. The *z* score informed on whether the upstream regulators were likely to be active or inhibited. A *z* score >2 and a robust *P* value (e.g., *P* < 1 × 10^−3^ or better) were required to consider an association as being significant and, thus, potentially of biological interest.

### Statistical analysis

All nonmicroarray statistics were carried out with the use of GraphPad Prism software (version 6.0; GraphPad Software). For the RT-qPCR analysis, a Mann-Whitney *U* test was performed with the use of a Δcycle threshold value between the control group and the rosiglitazone group. For the murine analysis, an unpaired *t* test was performed with the use of linear-expression values from exon-specific probe sets between control and rosiglitazone groups. For the human analysis, a paired *t* test was performed on values of total body weight, fat mass, and linear expression from probe sets before and after the intervention. Pearson correlation coefficients were derived between the change in weight and the change in gene expression with the intervention.

## RESULTS

**FIGURE 1:**
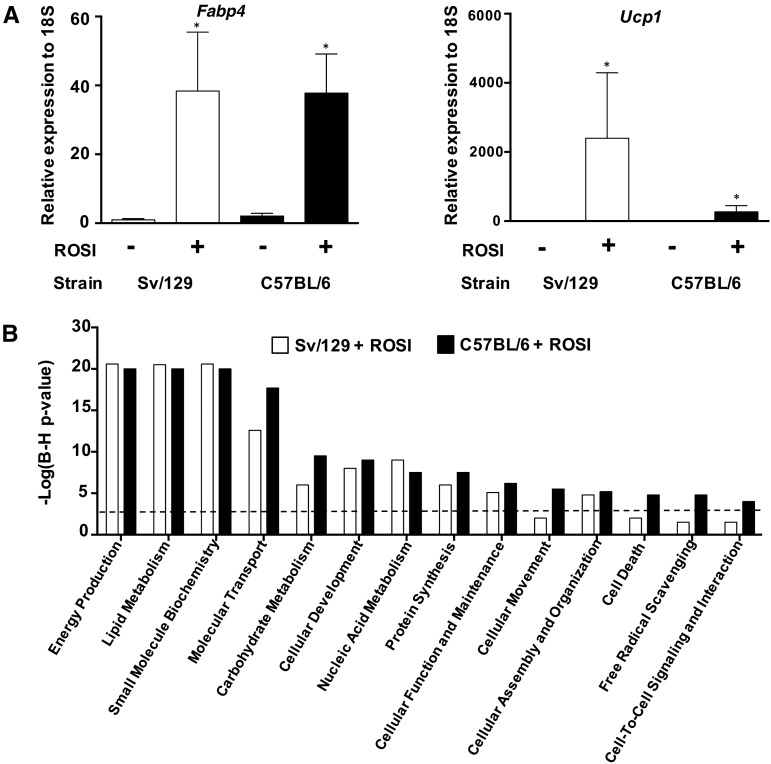
Characterization of the biology of the ROSI response between Sv/129 and C57BL/6 mice. (A) Mean ± SD RT-qPCR–derived expression changes of adipogenesis marker *Fabp4* and thermogenesis marker *Ucp1*; both genes were measured in inguinal white adipocytes originating from either Sv/129 or C57BL/6 mice, which were treated with or without ROSI and resulted in the following 4 groups: Sv/129 + ROSI, Sv/129 – ROSI, C57BL/6 + ROSI, and C57BL/6 – ROSI. The response from both strains confirmed the induction of browning and the maturation of adipocytes. Each group is composed of *n* = 4. *Significance between control and ROSI groups, *P* < 0.05. (B) Significance analysis of microarray was performed on Affymetrix Mouse Exon ST 1.0 (Affymetrix) with the use of RNA independent from the RT-qPCR experiment (Sv/129 + ROSI: *n* = 4; Sv/129 – ROSI: *n* = 4; C57BL/7 + ROSI: *n* = 4; and Sv/129 – ROSI: *n* = 5) and identified differentially expressed genes during brite formation in both strains. The gene lists from both strains were enriched in very similar pathways that were expectedly associated with metabolism (and thus, the majority of the analysis henceforth relied on the combination of both strains). The black dotted line represents the threshold for significance (B-H–corrected *P* = 1 × 10^−3^). B-H, Benjamini-Hochberg–corrected; *Fabp4*, fatty acid binding protein 4; ROSI, rosiglitazone; RT-qPCR, reverse transcriptase–quantitative polymerase chain reaction; *Ucp1*, uncoupling protein 1.

The rosiglitazone treatment resulted in substantial differential gene expression (**Supplemental List 1**). In C57BL/6-derived white adipocytes, the rosiglitazone treatment resulted in 1270 and 736 genes being upregulated and down regulated, respectively. In Sv/129 adipocytes, 833 and 308 genes were upregulated and downregulated, respectively. RT-qPCR validation confirmed similar *Fabp4*- and *Ucp1*-expression changes in both strains (**Figure 1A**) and the expected pathways that are associated with lipid metabolism and substrate oxidation in both strains (Figure 1B). An upstream analysis (**Supplemental Table 2**) showed that both Sv/129 and C57BL/6 gene lists were dominated by an extremely strong rosiglitazone-related transcriptional signature (Sv/129: *z* = 5.8, *P* = 1.2 × 10^−23^; C57BL/6: *z* = 6.5, *P* = 3.7 × 10^−30^) that was consistent with a PPARγ-activation signature (Sv/129: *z* = 5.7, *P* = 1.63 × 10^−25^; C57BL/6: *z* = 5.9, *P* = 4.16 × 10^−29^). This analysis provided robust experimental validation of the upstream-analysis method for subsequent use with the human clinical data.

**FIGURE 2:**
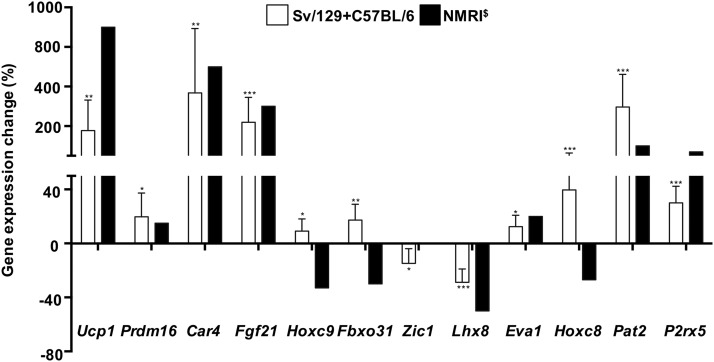
Establishment of reliable brite markers. The Affymetrix Exon ST 1.0 (Affymetrix) arrays that were used for pathway enrichment (Figure 1B) also provided exon-level changes that occurred with ROSI in detectable genes and were used to extract specific expression values corresponding to primers used in de Jong et al. (15) to calculate the mean ± SD percentage of change in expression in response to ROSI (*n* = 9) from the control (*n* = 8). All phenotypic adipocyte markers except for *Cidea* [which is not a valid adipocyte subtype marker in humans (9)] were measured. Only phenotypic adipocyte markers that reached significance are shown, whereas remaining phenotypic adipocyte-marker data are shown in **Supplemental Figure 1**A. ^$^Expression changes from de Jong et al. (15) are shown and were estimated from the original publication that used NMRI mice (15), which indicated that not all brite markers share the same directionality across studies. Student’s unpaired *t* test was performed with the use of linear expression intensities. *^,^**^,^***Significance: *P < 0.05, **P < 0.01, ***P < 0.001. *Car4*, carbonic anhydrase 4; *Eva1*; epithelial V-like antigen 1; *Fbxo31*, F-box protein 31; *Fgf21*, fibroblast growth factor 21; *Hoxc8*, Homeobox C8; *Hoxc9*, Homeobox C9; *Lhx8*, LIM homeobox protein 8; NMRI, Naval Medical Research Institute; *Pat2*, proton/amino acid transporter 2; *Prdm16*, PR domain 16; *P2rx5*, purinergic receptor P2X 5; *Ucp1*, uncoupling protein 1; *Zic1*, zinc finger protein of the cerebellum 1.

Various brite phenotypic biomarkers along with other genes measured by de Jong et al. (15), which relied on NMRI mice, were shown to be ∼60% consistent with Sv/129 and C57BL/6 mice (**Supplemental Table 3**). In our study, 12 of the biomarkers were significantly altered with rosiglitazone, but not all biomarkers were regulated in the same direction as noted in NMRI mice [e.g., homeobox C9 (*Hoxc9*), F-box protein 31 (*Fbxo31*), zinc-finger protein of the cerebellum 1 (*Zic1*), and homeobox C8 (*Hoxc8*) (in the current study, *Zic1* decreased with rosiglitazone] (**Figure 2**). Several additional phenotypic marker genes did not respond to rosiglitazone, including cluster of differentiation 137 (*Cd137*), transmembrane protein 26 (*Tmem26*), and cbp/p300-interacting transactivator 1 (*Cited1*) (Supplemental Figure 1A) indicating that they may be markers of cell origin. In addition, the exon-specific RNA profiling of LIM homeobox 11 (*Lhx8*), purinergic receptor P2X 5 (*P2rx5*), and alanine-serine-cysteine 1 (*Asc1*) showed that an interpretation of rosiglitazone’s effects was dependent on which exon was selected for the RT-qPCR analysis (**Supplemental Figure 2**). An analysis of house-keeping gene responses across strains (15) showed that this dependence did not explain differences across studies (Supplemental Figure 1B).

**FIGURE 3:**
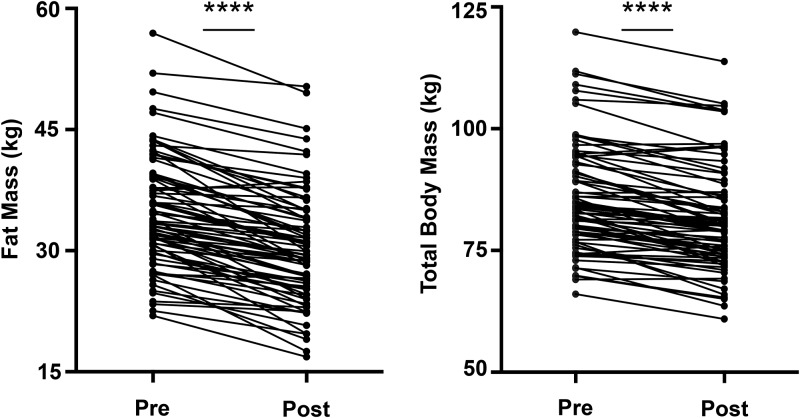
Effects of 16 wk of exercise and dieting on body composition. Subjects from Josse et al. (18, 19) were all premenopausal and overweight or obese women, and an overview of clinical characteristics is shown in Supplemental Table 2. Body-composition data (measured with the use of dual-energy X-ray absorptiometry) from each subject (with complete biopsies and body-composition data) were compared before and after the intervention and led to significant fat mass loss (A) and total body weight loss (B). Data are shown for all subjects (*n* = 79). Each dot and its respective connected dot represent data from a single subject. Student’s paired *t* test was performed; ****P < 0.0001.

**FIGURE 4:**
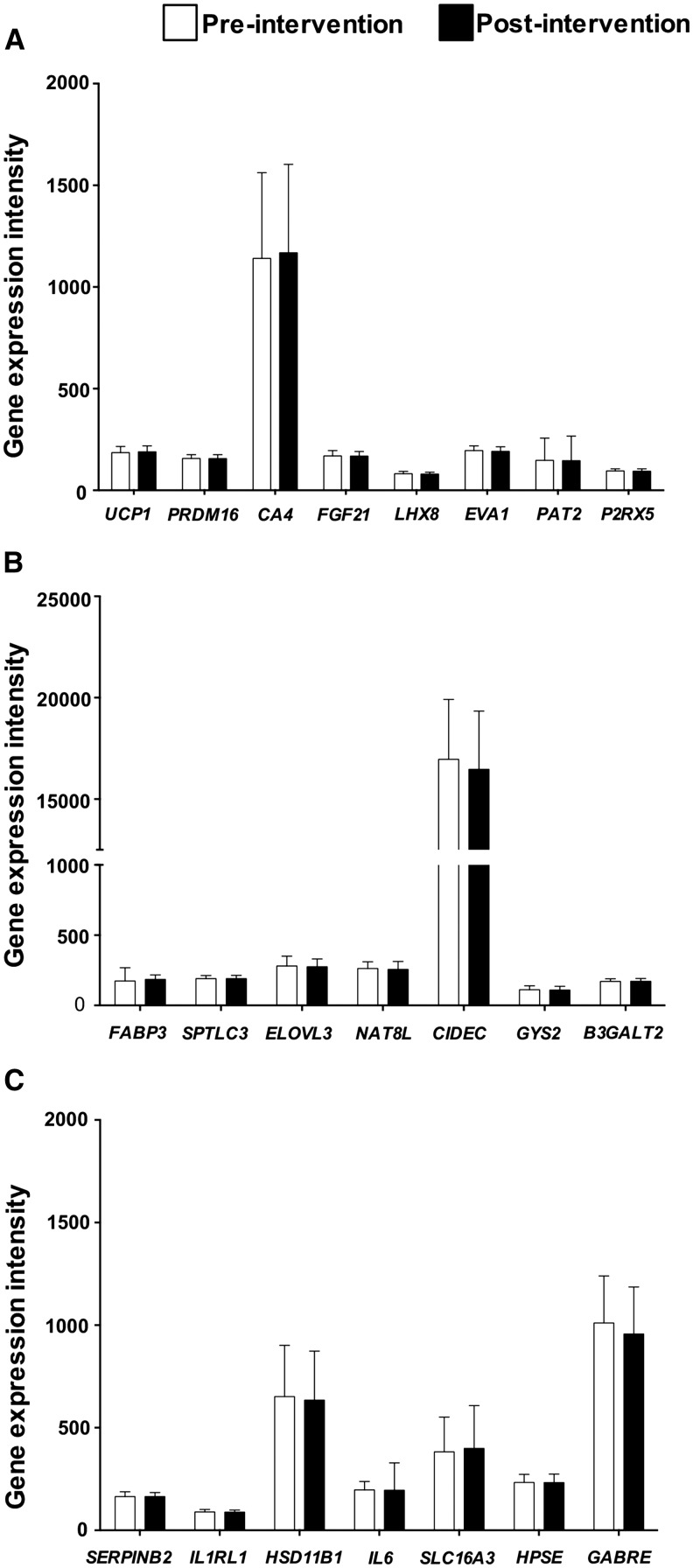
Evaluation of reliable brite markers and ROSI-regulated genes in white adipose tissue in response to exercise and dieting. HumanHT-12 V4.0 Expression BeadChip (Illumina) were used to measure gene-expression changes in 79 individuals before and after a 16-wk intervention. The data set was used to extract mean ± SD linear expression intensities corresponding to brite markers (A) that were consistent across Sv/129, C57BL/6, and NMRI mice, but no changes were shown. Note that *CA4* is the human ortholog of *Car4*. ROSI-regulated genes were measured in human subcutaneous white adipose tissue, but neither the most-upregulated ROSI genes (B) nor the most-downregulated ROSI genes (C) changed with intervention. Note that not all ROSI-responsive genes identified in mice had one-to-one orthologs, and certain Illumina probes hit multiple genomic regions, and thus, genes that fall into either of these categories were not included. *n* = 79 paired samples. Student’s paired *t* test was performed with the use of linear expression intensity (AU), but no significance was shown. *B3GALT2*, beta-1,3-galactosyltransferase 2; *CA4*, carbonic anhydrase 4; *CIDEC*, cell death inducing DFFA like effector c; *ELOVL3*, fatty acid elongase 3; *EVA1*, epithelial V-like antigen 1; *FABP3*, fatty acid binding protein 3; *FGF21*, fibroblast growth factor 21; *GABRE*, gamma-amiobutyric acid type A epsilon subunit; *GYS2*, glycogen synthase 2; *HPSE*, heparanase; *HSD11B1*, hydroxysteroid 11-beta dehydrogenase 1; *IL6*, interleukin 6; *IL1RL1*, interleukin 1 receptor like 1; *LHX8*, LIM homeobox 8; *NAT8L*, N-acetyltransferase 8 like; NMRI, naval medical research institute; *PAT2*, proton/amino acid transporter 2; *PRDM16*, PR domain containing 16; *P2RX5*, purinergic receptor P2X 5; ROSI, rosiglitazone; *SERPINB2*, serpin family B member 2; *SLC16A3*, solute carrier family 16 member 3; *SPTLC3*, serine palmitoyltransferase, long chain base subunit 3; *UCP1*, uncoupling protein 1.

The stringent biomarker genes, which reflected the exon-level analysis of the in vitro brite-formation studies, were checked to ensure comparability with the 3′ end of the same transcripts (data not shown) because the responses of these genes in human scWAT were measured with the use of a standard 3′ DNA microarray. As previously published (18, 19), there were substantial improvements in body composition after this gold-standard lifestyle intervention with a loss of fat mass (−4.4 ± 2.8 kg; *P* < 0.0001) and total body mass (−4.4 ± 3.5 kg; *P* < 0.0001) (**Figure 3**). However, we showed no evidence for the positive regulation of any brite marker gene in human scWAT in response to exercise and dieting (*P* > 0.05) (**Figure 4A**).

**FIGURE 5:**
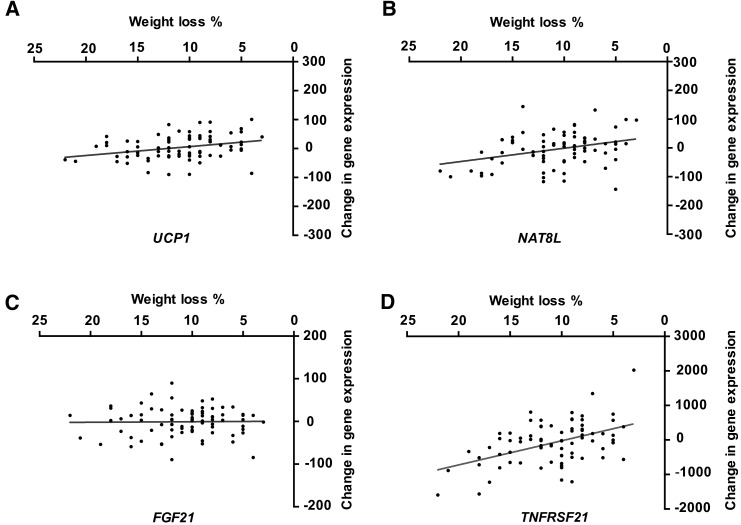
Relation between brite markers or rosiglitazone-responsive genes with combined exercise- and diet-induced weight loss. Of the 22 genes that were assessed (Figure 4) only *UCP1* (*P* = 0.006, *R*^2^ = 0.09) (A) and *NAT8L* (*P* = 0.003, *R*^2^ = 0.11) (B), a brite marker and a rosiglitazone up-regulated gene, respectively, showed a significant correlation between weight loss and a gene-expression change. In addition, both of these correlations went in the opposite direction because they both decreased with weight loss, which was the opposite effect if brite formation occurred. Another popular gene that is associated with brite formation is *FGF21* (*P* = 0.89, *R*^2^ = 0.0002) (C), but it showed no link with weight loss, whereas *TNFRS21* (*P* < 0.0001, *R*^2^ = 0.23) (D), which was identified with the use of quantitative significance analysis of microarray along with 180 other genes, showed a much-stronger correlation, implying that molecular changes are occurring with weight loss and are not linked to brite formation, at least in human subcutaneous white adipose tissue. A Pearson correlation was carried out between the change in linear expression intensity (AU) and the percentage of change in weight loss from paired samples with the use of subjects considered in the creation of Figure 4 (*n* = 79), and significance was determined at *P* < 0.05. *FGF21*, fibroblast growth factor 21; *NAT8L*, N-acetyltransferase 8 like; *TNFRSF21*, tumor necrosis factor receptor superfamily, member 21; *UCP1*, uncoupling protein 1.

In addition, the human adipose tissue gene-expression response to exercise training and calorie restriction was not related to the pattern of rosiglitazone-regulated genes (Figure 4B, C). When we correlated the extent of weight loss with changes in selected brite markers or rosiglitazone-regulated genes, we showed that *UCP1* (*P* = 0.006, *R*^2^ = 0.09) (**Figure 5A**) and N-acetyltransferase 8 like (*NAT8L*) (*P* = 0.03, *R*^2^ = 0.11) (Figure 5B) were decreased with greater weight loss (i.e., the opposite direction that was expected if brite formation was occurring), whereas fibroblast growth factor 21 (*FGF21*) showed no correlation (Figure 5C).

**FIGURE 6:**
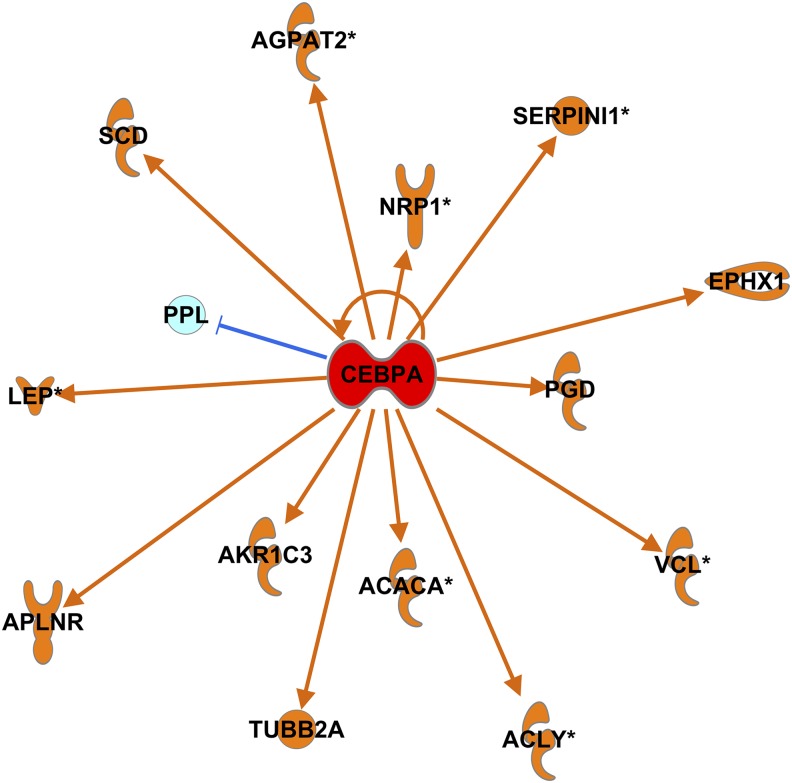
Upstream regulatory analysis of weight-loss–associated genes identified strong C/EBPα activation. With the use of 161 genes that were correlated with weight loss, their respective Pearson correlation coefficients were used in an ingenuity pathway analysis: upstream analysis, which identified potential regulators that either are responsible or mimic the effect of exercise- and diet-induced weight loss (Supplemental Table 4). A strong activation of C/EBPα (*z* score = 2, *P* = 6.6 × 10^−7^) occurred, which led to numerous downstream signalings that ultimately affected adipogenesis. *Significantly regulated by C/EBPα in multiple species. ACACA, acetyl-CoA carboxylase alpha; ACLY, ATP citrate lyase; AGPAT2, 1-acylglycerol-3-phosphate O-acyltransferase 2; AKR1C3, aldo-keto reductase family 1, member C3; APLNR, apelin receptor; CEBPA, CCAAT/enhancer binding protein α C/EBPα, CCAAT/enhancer binding protein α EPHX1, epoxide hydrolase 1; LEP, leptin; NRP1, neuropilin 1; PGD, phosphogluconate; PPL, periplakin; SCD, stearoyl-CoA desaturase; SERPINI1, serpin family I member 1; TUBB2A, tubulin beta 2A class IIa; VCL, vinculin.

An unbiased DNA microarray analysis of human scWAT in response to the intervention showed 155 RNAs were positively correlated with weight loss and 26 RNAs were negatively correlated with weight loss (FDR <10%) (**Supplemental List 2**). An example of a gene that was univariately correlated with weight loss (*TNFRSF21)* is shown in Figure 5D (*R*^2^ = 0.22, *P* < 0.0001). After uploading the 181 genes and the detected background genes into the IPA database (**Supplemental List 3**), 161 genes were available for analysis. These weight-loss–correlated genes showed enrichment in the expected pathways such as lipid metabolism (**Supplemental Figure 3**) as well as evidence for dynamic tissue remodeling including adipogenesis rather than a simplistic atrophy of adipose tissue mass (**Supplemental Table 4**). For instance, we showed evidence for CCAAT/enhancer binding protein α (C/EBPα) activation (*z* = 2.0, *P* = 6.6 × 10^−7^) that was indicative of adipogenesis (**Figure 6**). We also noted a marginal result of interest (less than the stated *z* = 2 threshold) [i.e., adenosine A2 activation (*z* = 1.9, *P* = 7.9 × 10^−4^)] upstream of some of the transcriptional responses in scWAT during weight loss. More-robust results included evidence for liver X receptor α/β (LXRalpha/β) agonism (*z* = 2.1, *P* = 2.8 × 10^−7^) and the inhibition of leptin-like signaling (*z* = −2.6, *P* = 3.9 × 10^−5^).

## DISCUSSION

In the current study, we discovered that *UCP1* expression was negatively correlated with weight loss in humans in response to exercise training combined with modest energy restriction. Our data appear to rule out the hypothesis that exercise promotes weight loss through brite formation (10) at least in women and when a negative energy balance is induced via a combination of strategies. The study of the molecular drivers that regulate the WAT phenotype, including the promotion of the formation of brite adipocytes, is a complex process that requires both in vitro and in vivo models (24). Brite formation has been proposed as a mechanism to increase the metabolic rate to promote weight loss in obese subjects (10). Several factors limit the progress toward the testing of this hypothesis including a lack of novel drugs to stimulate brite formation. In addition, knowledge of reliable biomarkers for brite formation that are consistent in both preclinical and clinical models are needed (i.e., for drug-development studies).

The upstream analysis showed that rosiglitazone-treated cells displayed a clear pattern of gene expression that reflected PPARγ agonism (Supplemental Table 2). We also showed that the rosiglitazone transcriptional signature was strongly linked to mono-(2-ethylhexyl)phthalate (MEHP) activity (*z* = 5.6, *P* = 2.7 × 10^−31^). An environmental obesogen, MEHP originates from plastics and, after either in vitro or in utero exposure, promotes adipogenesis (25). The impact of MEHP on brite formation is unknown, but the presence of this common environmental factor complicates the interpretation of UCP1 expression analyses in adult humans. Mimicking global aspects of PPARγ agonism, MEHP has a PPAR co-activation activity (26, 27). Although phthalate metabolites have been associated with increased diabetes risk, MEHP has not (28), which suggests that a greater understanding of the molecular actions of MEHP may yield insight into how to safely promote brite formation.

We were able to identify a number of compounds that partially overlap with the brite gene-expression program. The P38 mitogen-activated protein kinase inhibitor SB203580 was significantly associated with the rosiglitazone signature (*z* = 4.69, *P* = 6.8 × 10^−14^) as was PD98059, which is an indirect extracellular signal-regulated kinase 1/2 (ERK1/2) inhibitor (*z* = 6.15, *P* = 2.1 × 10^−12^). Holstrom et al. (29) showed that Erk1/2 activation was involved in the initial proliferation response in brown preadipocytes. U0126, which is another indirect inhibitor of ERK1/2, which also appeared important for activating an adipogenic program in human mesenchymal stem cells (30). The global rosiglitazone transcriptional signature also overlapped with mitogen-activated protein kinase kinase kinase kinase 4 (MAP4K4) inhibition (*z* score = –5.05, *P* = 4.8 × 10^−27^). MAP4K4 was subject to a drug-discovery effort, and the silencing of Map4k4 induces *Myf5,* which is a developmental muscle and brown-adipocyte cell marker (31). We suspect that it is unlikely that MAP4K4 inhibitors have a sufficient safety profile to represent a chronic strategy for promoting brite formation (32). However, single-target drugs for complex chronic diseases are also unlikely, and it would be more appealing to consider combinatorial therapy options, e.g., combined ERK activation and MAP4K4 inhibition targeting distinct components of the rosiglitazone RNA-expression program.

Although a variation in the extent of the browning response in different scWAT depots is accepted, the issue of variation can be extended by consideration of both the genetic background (33) and transcript variants (the exon targeted for RT-qPCR) of each phenotypic marker. Some phenotypic markers have an unclear biological role during browning, whereas some markers such as *Fgf21* are thought to actively influence thermogenesis (34). The adipocyte markers described in the current study are representative of markers that have been commonly used in literature (15). One recent study claimed to determine the stringency of brite markers with the use of NMRI mice (15). We revisited these data (15) (Supplemental Table 3), and in general terms (directionality), there was only an ∼60% agreement between studies. Although the use of different messenger RNA quantification methods (RT-qPCR compared with microarray) can introduce differences, exon arrays allowed us to match the exon and housekeeping-gene analyses across studies (Supplemental Figure 1B). However, with the use of a subset of stringent brite biomarkers, we showed no regulation in adipose women in a negative–energy-balance state. The current repertoire of murine-derived phenotypic adipocyte markers may need to be superseded by human studies; however, efforts to profile human adipose tissue (35) must control for the presence of contaminating cell types (i.e., blood, blood vessels, and nerves). There are also limitations of our in vitro models (2, 20, 21) because we do not fully capture the complexity of brite formation or adipose tissue dynamics in vivo, and thus, specific markers in the context of an in vitro system can become nonspecific in a human tissue biopsy.

Increasingly, it has become accepted that chronic exercise training should alter the phenotype of adipose tissue and not just reduce its mass (10). If exercise training induced greater UCP1 activity, this effect might represent a counterintuitive increased capacity for heat generation (rather than dissipation). In contrast, excessive adipose tissue loss reduces the role of human adipose tissue in thermal insulation, and perhaps a greater thermogenic capacity would have been beneficial for survival (but now we have clothes and central heating). Nevertheless, we observed that human scWAT samples did not show signs of browning with 16 wk of exercise training despite marked improvements in fitness, fat loss, and the identification of specific molecular programs that are related to the remodeling of adipose tissue. We noted a decrease in *UCP1* expression with weight loss, which was perhaps an adaptive response (whitening) that reflected reduced calorie intake or a negative energy balance. We note that *UCP1* expression can be lower in leaner individuals (9), which suggests that our finding is not paradoxical, at least in humans. However, a potential limitation of this study was the lack of a comparative energy-restriction-only group to determine the contribution of reduced calorie intake on scWAT. In addition, our study consisted of women, and we only assessed one scWAT region.

Emerging studies on WAT biology have been primarily focused on understanding mechanisms that transform WAT to energy-dissipating tissue (10). However, a greater understanding of molecular adaptations concomitant with weight loss will provide insights in understanding WAT plasticity and health. The loss in adipose mass with exercise has been commonly reported to reflect reduced adipocyte size (36), and whether the adipocyte number changes is not often reported with exercise. Our molecular network analysis showed evidence that there was increased C/EBPα activity in the tissue, which was concurrent with exercise- and diet-induced weight loss, and such activity is typically associated with adipogenesis. Therefore, it would seem that our intervention may have increased adipocyte turnover because the total adipocyte number remains roughly stable with weight loss (37).

We showed evidence for the inhibition of local adipose leptin signaling as well as LXRα/β agonism. Previous studies have showed that, when human white adipocytes are treated with an LXR agonist, there is an increased lipolysis and β oxidation (38). With the use of an IPA, we identified an intriguing overlap with 11 genes that we previously showed (39) were related to the ability to remodel the cardiovascular capacity, thereby suggesting that these adaptability genes may influence the potential to remodel multiple tissue (4 11 genes; Benjamini-Hochberg–corrected *P* = 5 × 10^−3^).

In conclusion, with the use of a genuinely stringent repertoire of brite biomarkers in human scWAT samples, 16 wk of exercise training with calorie restriction does not result in increased *UCP1* expression or of any other brite biomarker despite evidence for the molecular remodeling of adipose tissue.

## Footnotes

9 Abbreviations used: ERK, extracellular signal-regulated kinase; FDR, false discovery rate; IPA, ingenuity pathway analysis; LXR, liver X receptor; MAP4K4, mitogen-activated protein kinase kinase kinase 4; MEHP, mono-(2-ethylhexyl)phthalate; NMRI, Naval Medical Research Institute; PPARγ, peroxisome proliferator–activated receptor-γ RT-qPCR, reverse transcriptase–quantitative polymerase chain reaction; scWAT, subcutaneous white adipose tissue; UCP1, uncoupling protein 1; WAT, white adipose tissue.
